# Supplementary material for: Plasmonic metasurfaces of cellulose nanocrystal matrices with quadrants of aligned gold nanorods for photothermal anti-icing
Source: Nat Commun. 2023 Dec 8;14:8096. doi: 10.1038/s41467-023-43511-9 (PMC10709361; doi:10.1038/s41467-023-43511-9)
Supplement: Supplementary file 1 — Supplementary Information [file 41467_2023_43511_MOESM1_ESM.pdf]

Supplementary Information

Plasmonic Metasurfaces of Cellulose Nanocrystal Matrices with Quadrants of Aligned Gold Nanorods for Photothermal Anti-Icing

Jeongsu Pyeon[1,5†], Soon Mo Park[2,3,5†], Juri Kim[3], Jeong-Hwan Kim[1], Yong-Jin Yoon[1], Dong Ki Yoon[3,4*], and Hyoungsoo Kim[1*]

[1]Department of Mechanical Engineering, Korea Advanced Institute of Science and Technology, Daejeon 34141, Republic of Korea
[2]Department of Chemical and Biomolecular Engineering, Cornell University, Ithaca, NY, 14853, USA
[3]Graduate School of Nanoscience and Technology, Korea Advanced Institute of Science and Technology, Daejeon 34141, Republic of Korea
[4]Department of Chemistry and KAIST Institute for NanoCentury, Korea Advanced Institute of Science and Technology, Daejeon 34141, Republic of Korea
[5]These authors contributed equally: Jeongsu Pyeon, Soon Mo Park
[*]e-mail: nandk@kaist.ac.kr; hshk@kaist.ac.kr

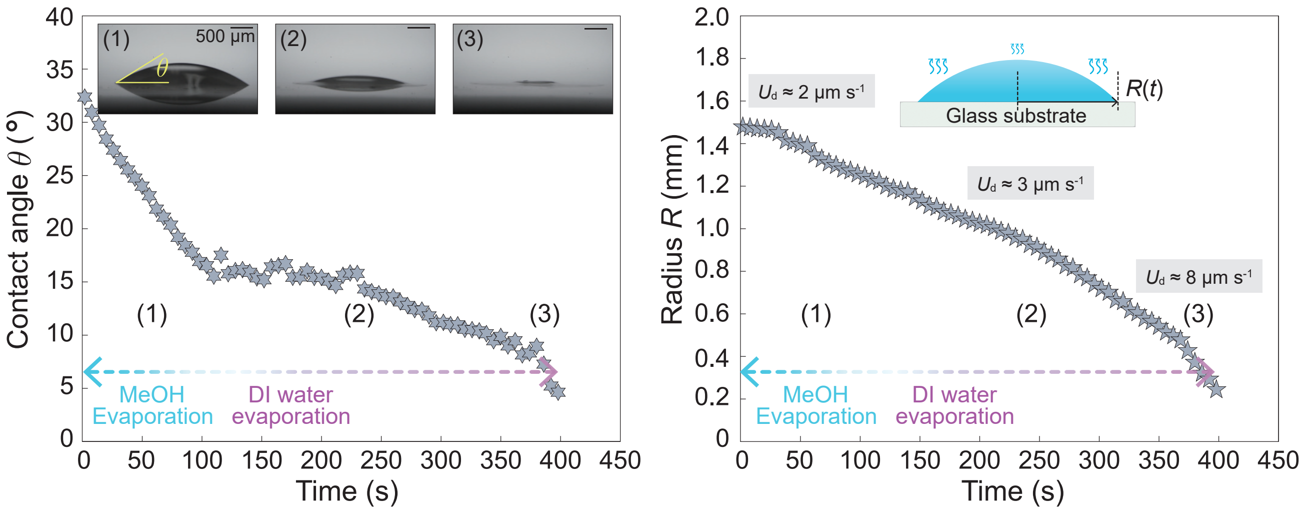

Supplementary Figure 1. Measurement results of time-dependent contact angle (θ) and the radius (R) of CNC dispersed evaporating droplet. A 2 μL droplet was deposited onto a glass substrate with a coating solution composed of 3.40 wt% CNC in a mixture of MeOH and DI water (= 62.75 : 33.85 wt%). **a** Evolution of the contact angle can be divided into three distinct regimes as follows: (1) The rapid evaporation of MeOH caused a sudden decrease in the contact angle in the early stage. (2) Afterward, the contact line of the droplet freely receded with little change in the contact angle ($\Delta\theta \approx 5°$). (3) Just before the end of the evaporation process, a thin film formed, as shown in the insets, and dried quickly (less than 30 s). **b** Time-dependent droplet radius changes during the evaporation process. The scale bars in the insets are 500 μm. Source data are provided as a Source Data file.

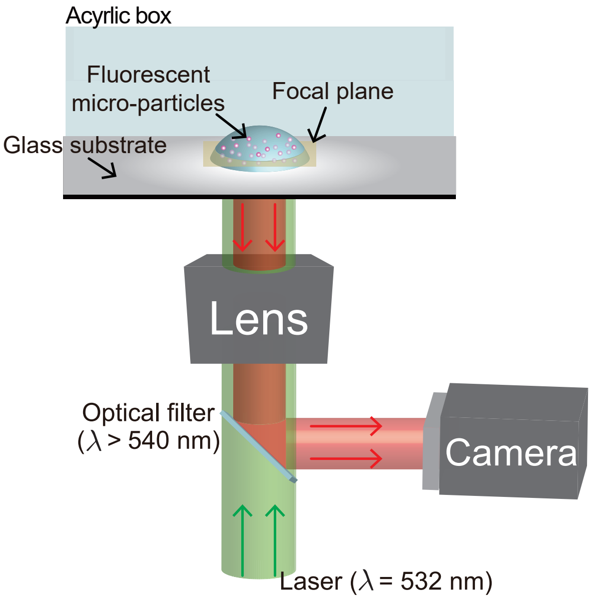

Supplementary Figure 2. Illustration of micro-particle image velocimetry (μ-**PIV).** Detailed setup information is described in the Methods section.

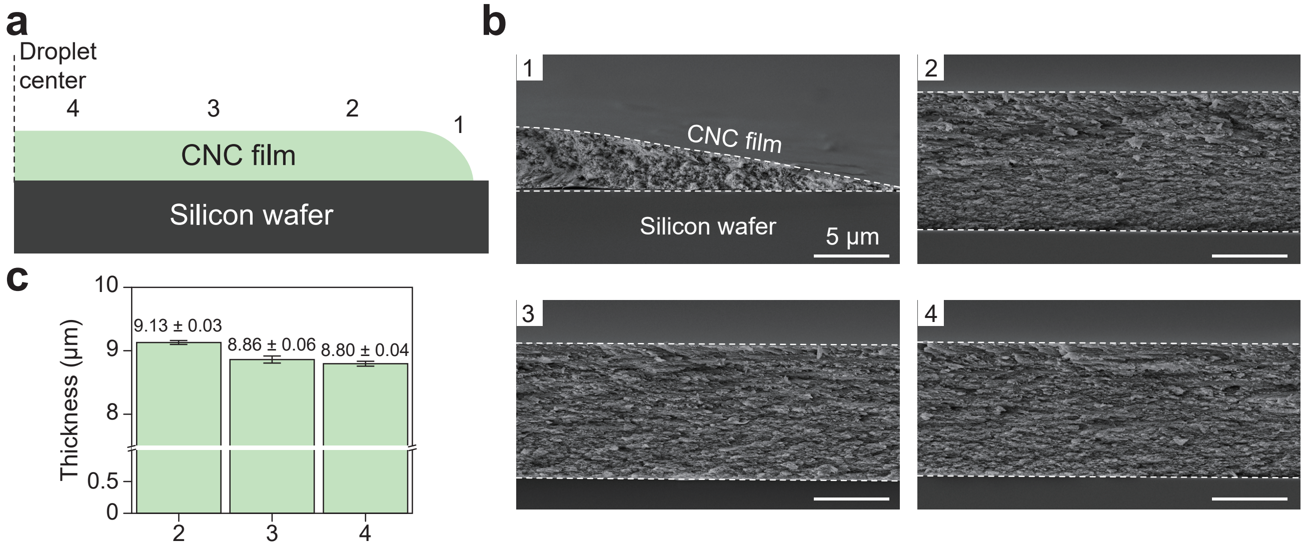

Supplementary Figure 3. Analysis of CNC film thickness using side-view Scanning Electron Microscope (SEM) images. a-b The side view of the CNC film was captured in sequential order from the vicinity of the droplet's edge to the vicinity of the droplet's center (1 - 4). **c** The average thickness was measured at each location. All error bars were obtained through multiple measurements conducted at least three times. All scale bars are 5 μm.

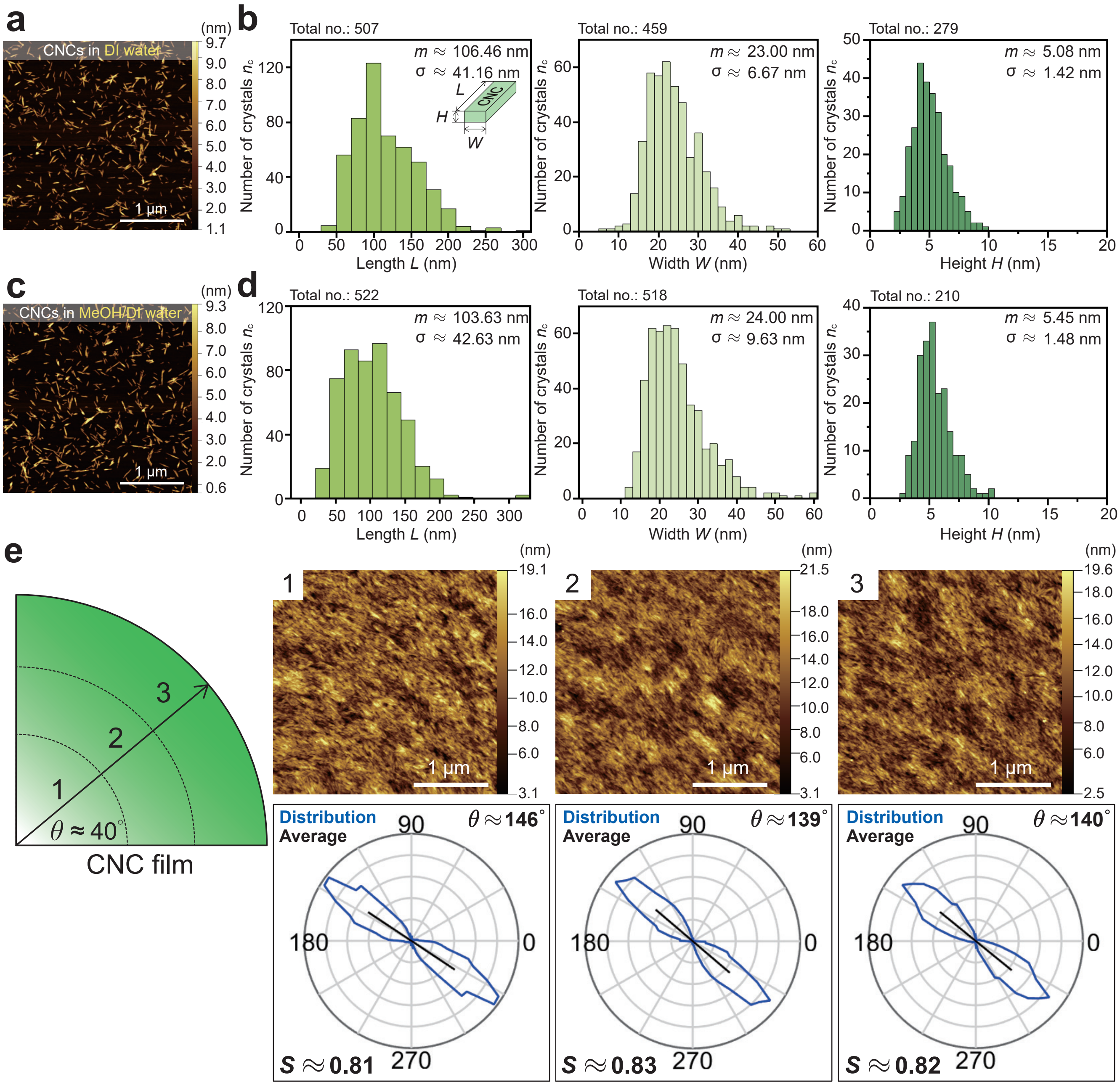

Supplementary Figure 4. Characterization of CNC particles and CNC films by Atomic Force Microscope (AFM). a Representative AFM images of CNCs dispersed in DI water. **b** Histogram of length, width, and height distribution of CNC particles in DI water. **c** Representative AFM images of CNCs dispersed in a MeOH and DI water mixture. **d** Histogram of length, width, and height distribution of CNC particles in a MeOH and DI water mixture. **e** AFM images (top) and rotation distribution (bottom) of the CNC film at (1) the inner, (2) the intermediate, and (3) the outer ring region in the direction of 40°. The CNC film was created by evaporating a droplet containing MeOH : DI water : CNCs = 62.75 : 33.85 : 3.40 wt%. The order parameter (*S*) indicates the degree of alignment of the CNCs. Source data are provided as a Source Data file.

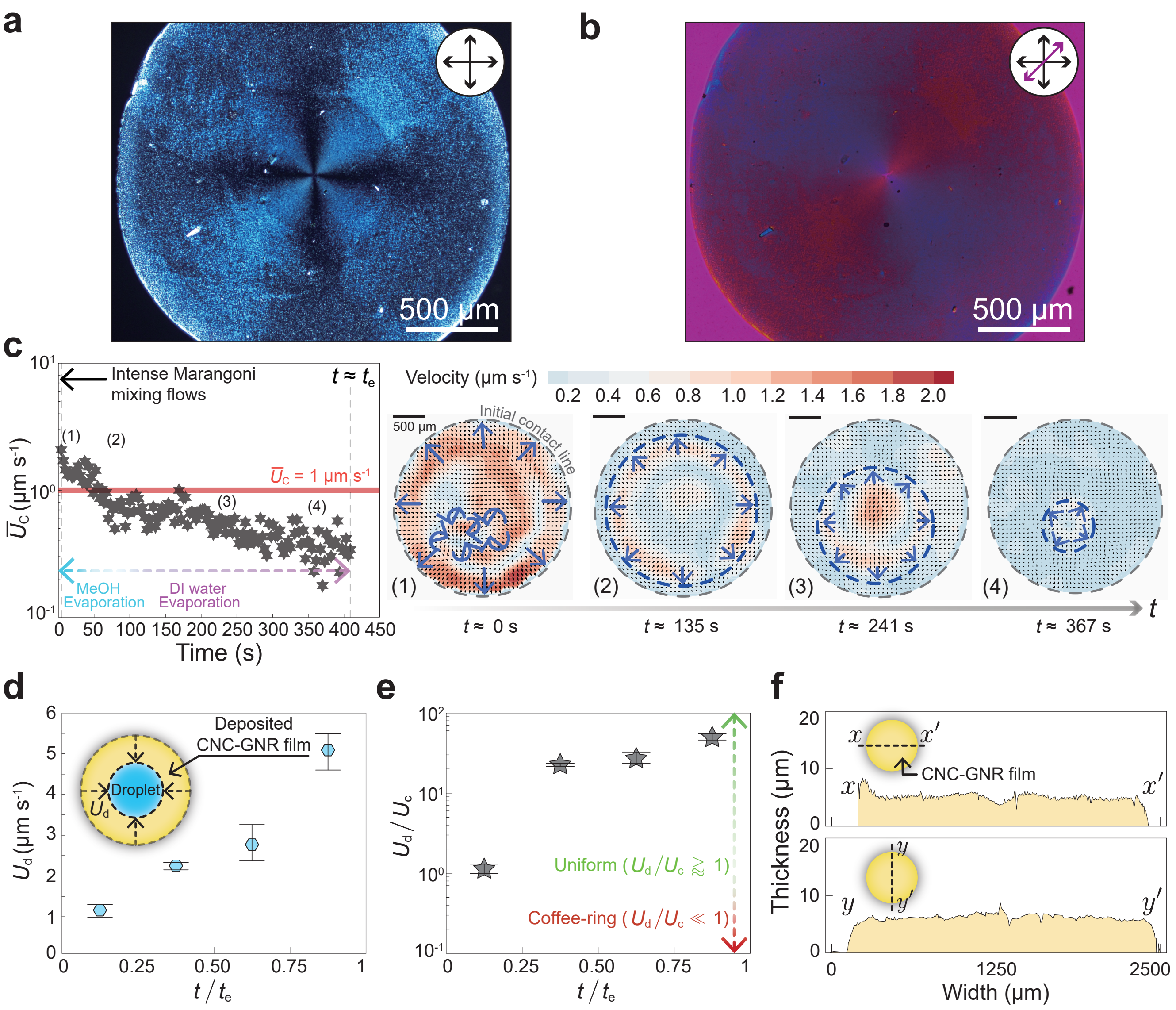

Supplementary Figure 5. Uniformly co-assembled GNRs in a quadrant CNC matrix. a POM (polarized optical microscopy) and **b** POM-r (with a retardation plate) images of the CNC-GNR dot film on a glass substrate. **c** Micro-particle image velocimetry (μ-PIV) results for the evaporation of droplets containing a mixture of 70% MeOH and 30% DI water by volume, with 3.40 wt% CNCs and 0.28 wt% GNRs, observed following a substantial reduction in solutal-Marangoni mixing flows. Typical flow structures were denoted by blue arrows, and the positions of moving contact lines were identified with blue dashed circles. t_e indicates the time at which the droplets were entirely evaporated. A detailed visualization of the flow pattern is provided in Supplementary Movie 7. **d** Time-dependent self-dewetting speed U_d of (**c**). **e** Speed analysis results of the dewetting speed U_d and coffee-ring speed U_c of (**a** and **b**). Each error bar was obtained from four samples conducted in separate experiments. **f** Film thickness profiles of (**a** and **b**) along the x-x' and y-y' lines. The scale bar indicates 500 μm. Source data are provided as a Source Data file.

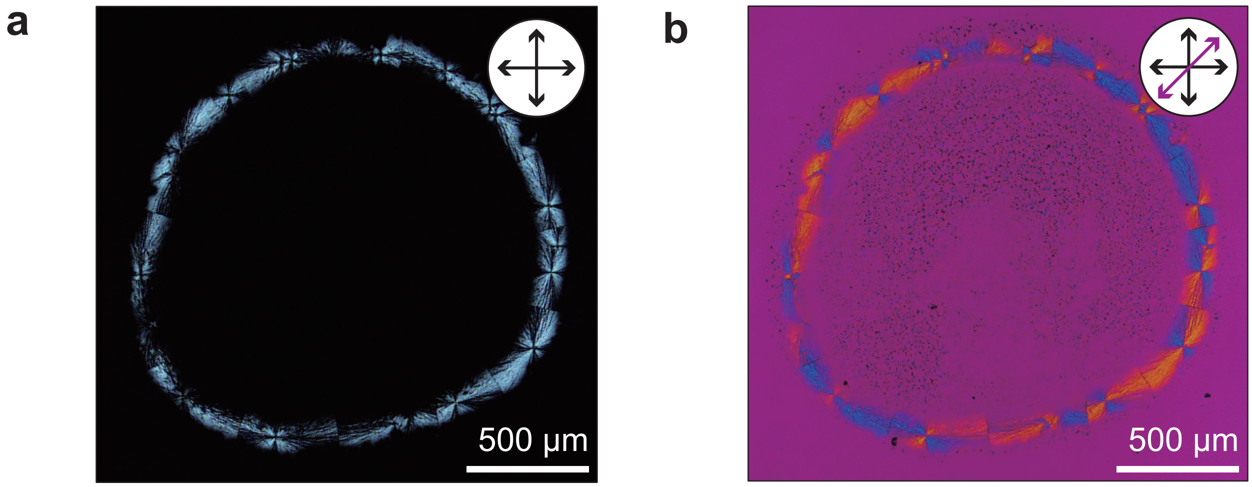

Supplementary Figure 6. Polarized optical microscopy (POM) images of GNR films obtained from the evaporation of a pure GNR dispersion droplet, without a CNC template. **a** POM and **b** POM-r (with a retardation plate) images were observed. The composition of the GNR solution was MeOH : DI water : GNRs = 64.88 : 35.00 : 0.12 wt%.

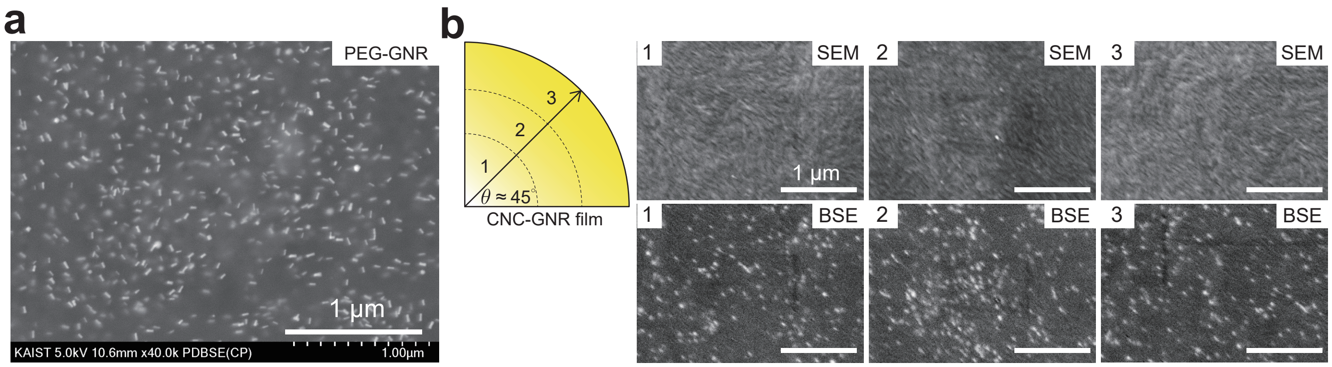

Supplementary Figure 7. Characterization of CNC-GNR films by Scanning Electron Microscope-Backscattered Electron (SEM-BSE). **a** Representative SEM images of PEG-GNR particles. **b** SEM (top) and BSE (bottom) images of the CNC-GNR film at (1) the inner, (2) the intermediate, and (3) the outer ring region in the direction of 45°. For the SEM-BSE measurements, the CNC-GNR film was deposited onto a silicon wafer substrate by evaporating the droplet composed of MeOH : DI water : CNCs : GNRs = 62.68 : 33.82 : 3.39 : 0.11 wt%.

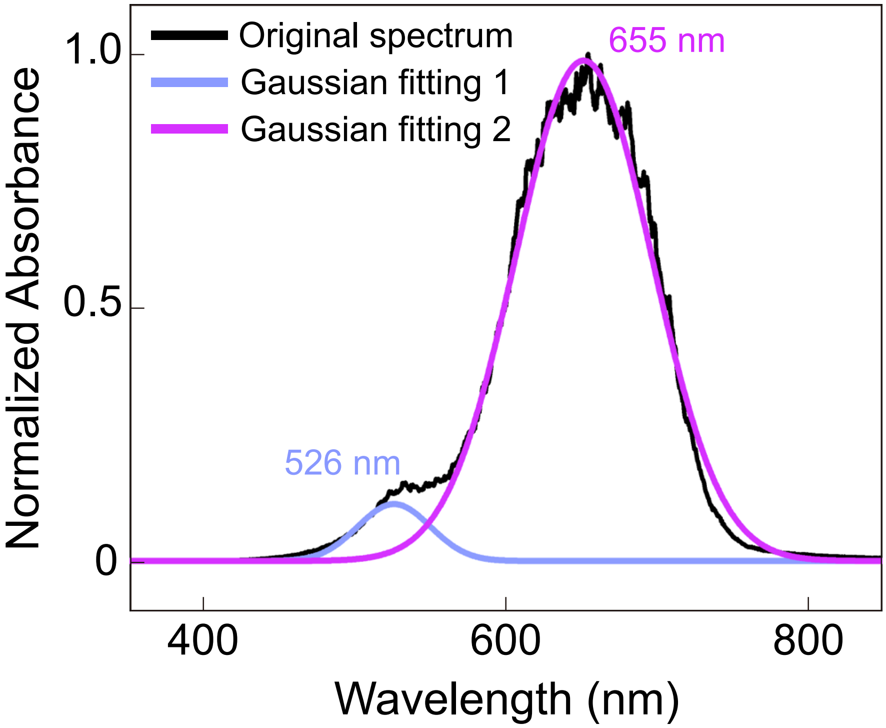

Supplementary Figure 8. Results of UV-Vis absorption spectra of a CNC-GNR film. The absorption spectrum of the film exhibited a distinct maximum peak along with a shoulder peak on the left side. By performing Gaussian fitting, two separate graphs were obtained with peak maxima at 526 nm (blue) and 655 nm (purple).

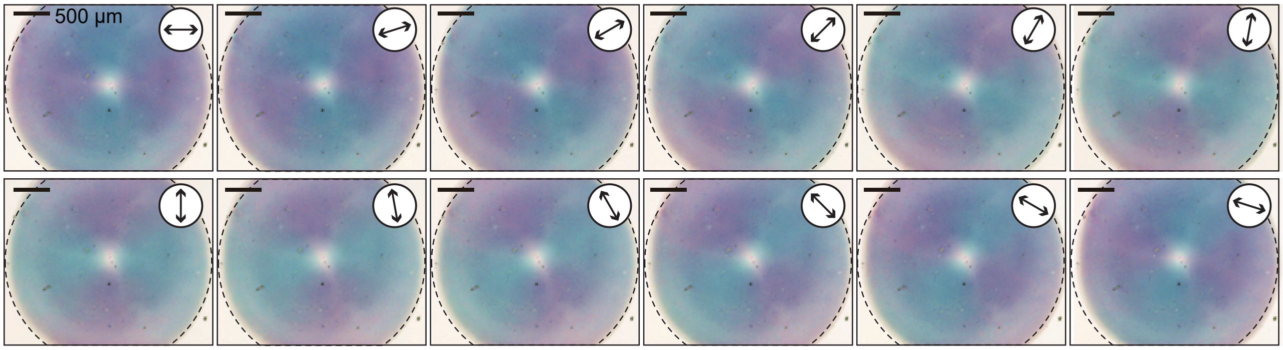

Supplementary Figure 9. Sequential snapshots of plasmonic color change depending on the polarization orientation. An optical microscope captured all images with a single linear polarizer rotating 180° (indicated by black double arrows). The images show two plasmonic optical colors (blue and light violet) observed due to the localized surface plasmon resonance (LSPR) of GNRs on a CNC-GNR film. The scale bar is 500 μm.

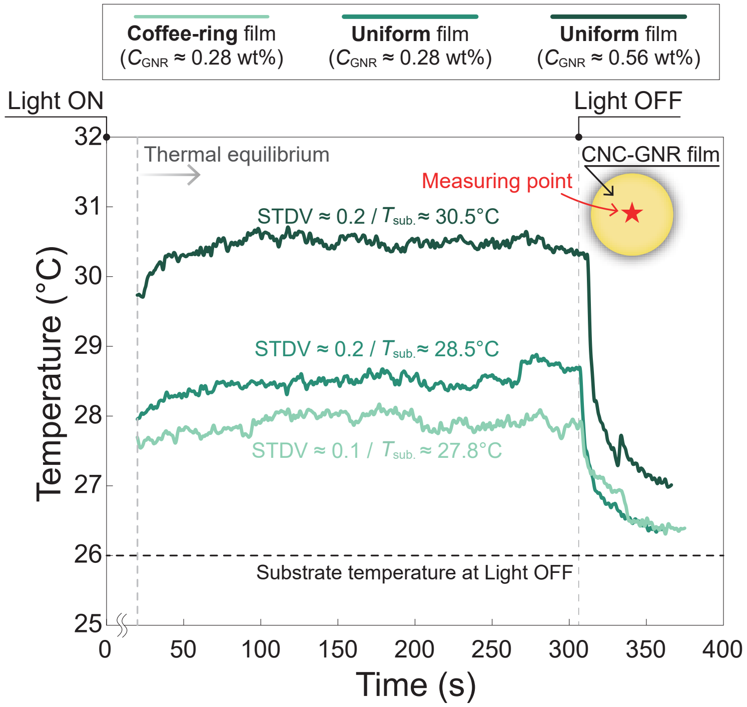

Supplementary Figure 10. Real-time point temperature measurement results near the center of the CNC-GNR film. The temperature data were compared depending on two parameters; (i) uniformity of the CNC-GNR films [a light greenish line vs. a greenish line] and (ii) GNR concentrations [a greenish line ($C_{GNR} \approx 0.28$ wt%) vs. a dark greenish line ($C_{GNR} \approx 0.56$ wt%). All data were plotted starting from the moment thermal equilibrium was almost achieved, which was approximately 20 s later. The standard deviations (STDV) and average temperatures (T_{avg}) were calculated based on measurement data ranging from 20 - 305 s. Source data are provided as a Source Data file.

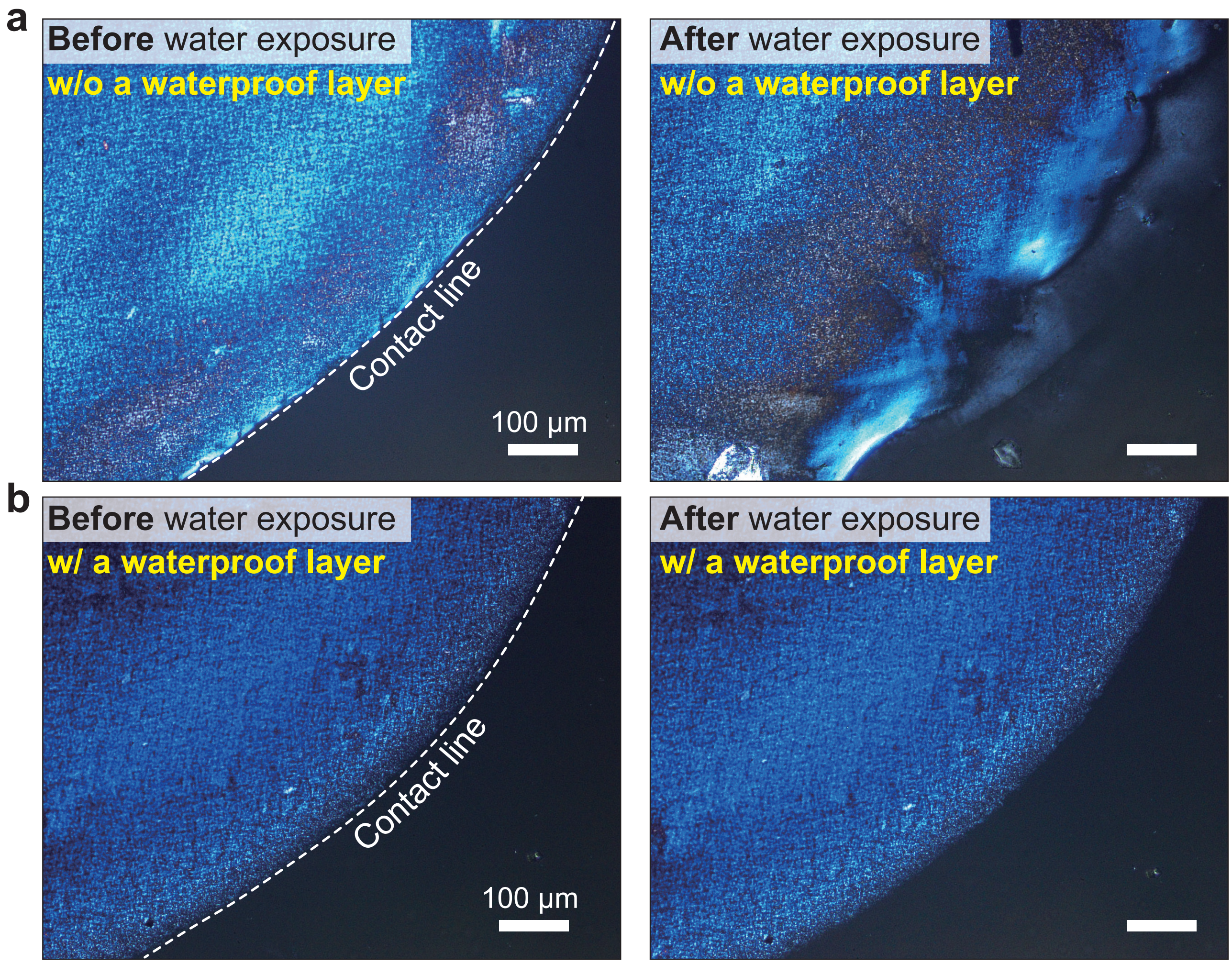

Supplementary Figure 11. Structural changes in CNC-GNR films upon water contact, without and with a waterproof layer. The CNC-GNR films (**a**) without and (**b**) with the waterproof layer were observed and compared using polarized optical microscopy. To prevent the structural changes of the co-assembled CNC-GNR from water, nail polish oil was brushed onto its surface and allowed to dry at room temperature to create a waterproof layer.

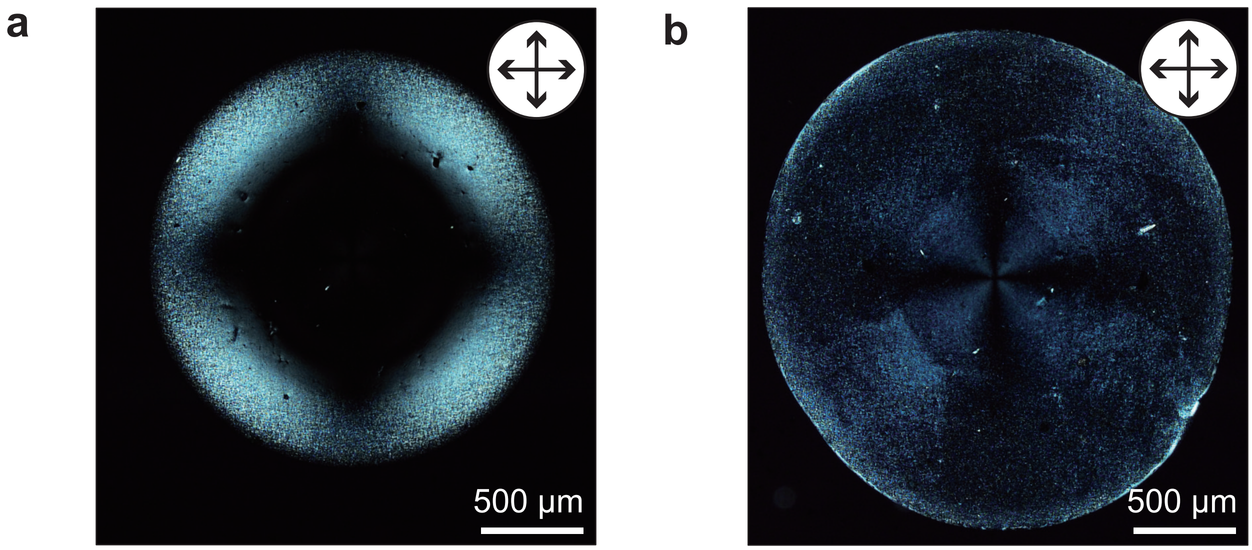

Supplementary Figure 12. Comparison between coffee-ring and uniform CNC-GNR films under POM measurements. The images show a comparison between **a** coffee-ring and **b** uniform CNC-GNR patterns obtained from 2 μL drop evaporation of a pure DI water (100 vol.%) and MeOH and DI water mixture (70 : 30 vol.%), respectively.

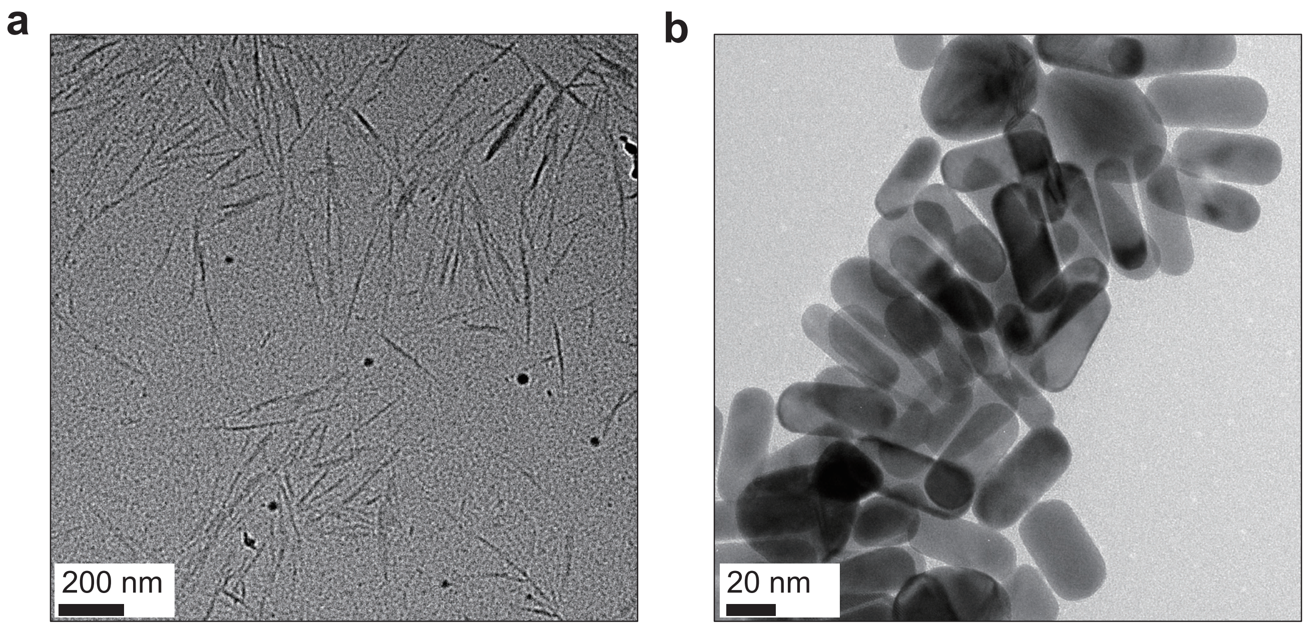

Supplementary Figure 13. Characterization of CNCs and GNRs by transmission electron microscopy (TEM). **a** Rod-shaped CNCs and **b** PEG-coated GNRs were observed by TEM measurements, respectively.

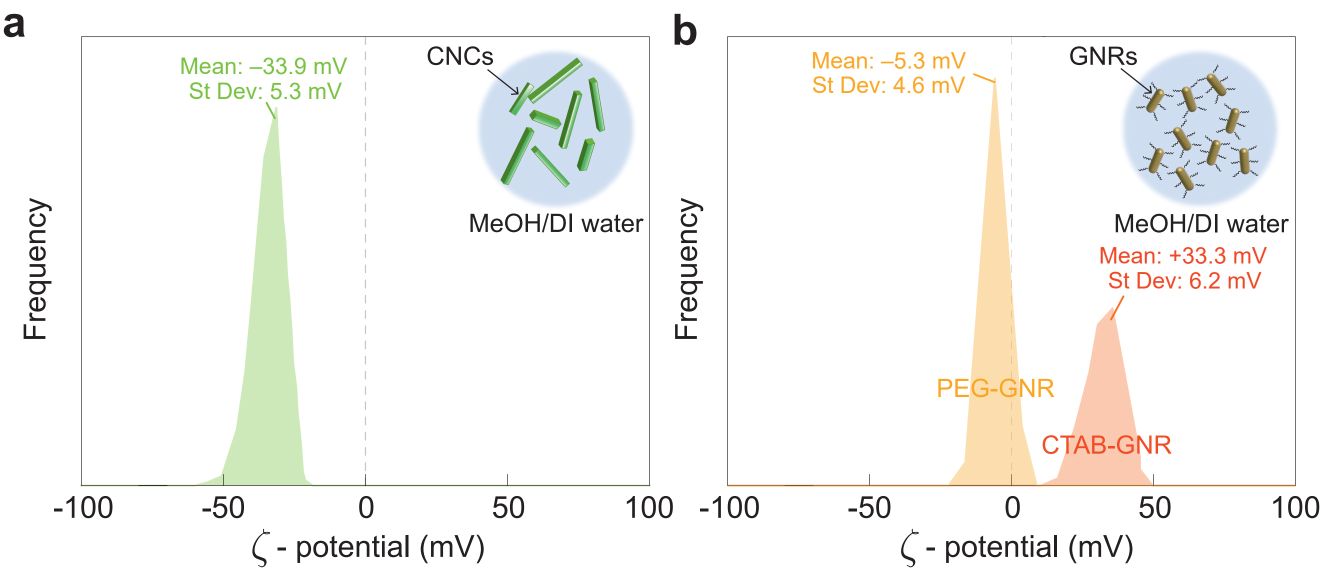

Supplementary Figure 14. Results of ζ-potential measurements of the (a) CNC and (b) GNR dispersions with a MeOH and DI water mixture. All particles were dispersed in the binary mixture containing 70% MeOH and 30% DI water, based on the volume ratio. The particle concentrations were approximately 1 mg/mL. Source data are provided as a Source Data file.

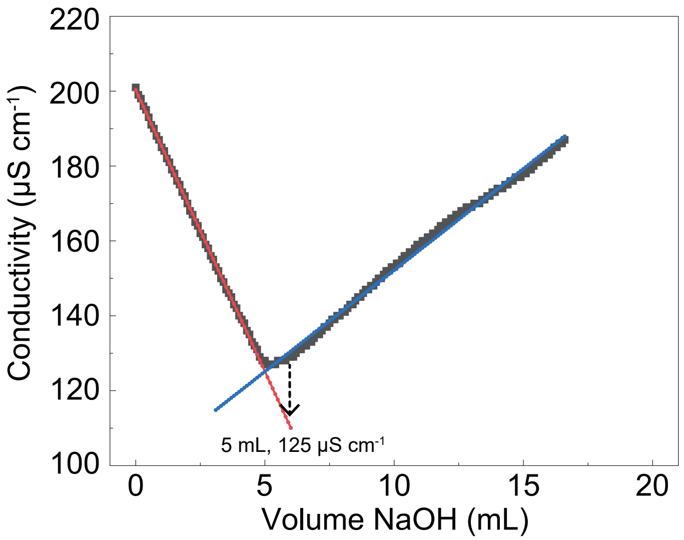

Supplementary Figure 15. Conductometric titration curves for protonated sulfate half-ester CNCs. The equivalent volume was estimated by the intersection of negative (red) and positive (blue) lines. Source data are provided as a Source Data file.

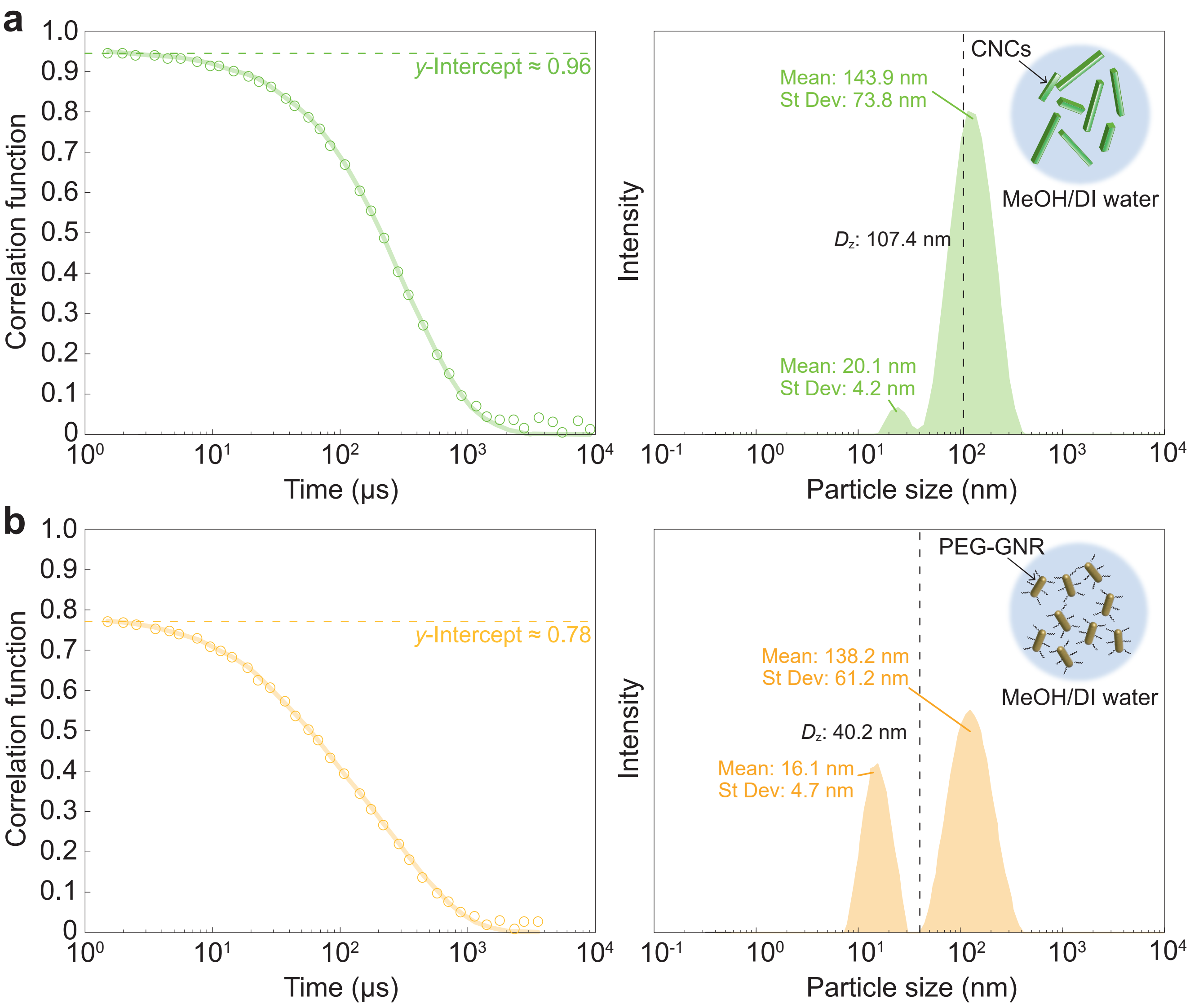

Supplementary Figure 16. Size distribution of CNC and PEG-GNR particles in a MeOH and DI water mixture. Dynamic Light Scattering (DLS) analysis was conducted to determine the size distribution of the particles and assess their colloidal stability in the binary mixture containing 70% MeOH and 30% DI water. The correlation functions (on the left) and intensity-based particle size distributions (on the right) of (**a**) CNC and (**b**) PEG-GNR particles were measured. D_z represents the z-average hydrodynamic diameter. The particle concentrations were approximately 1 mg/mL. Source data are provided as a Source Data file.

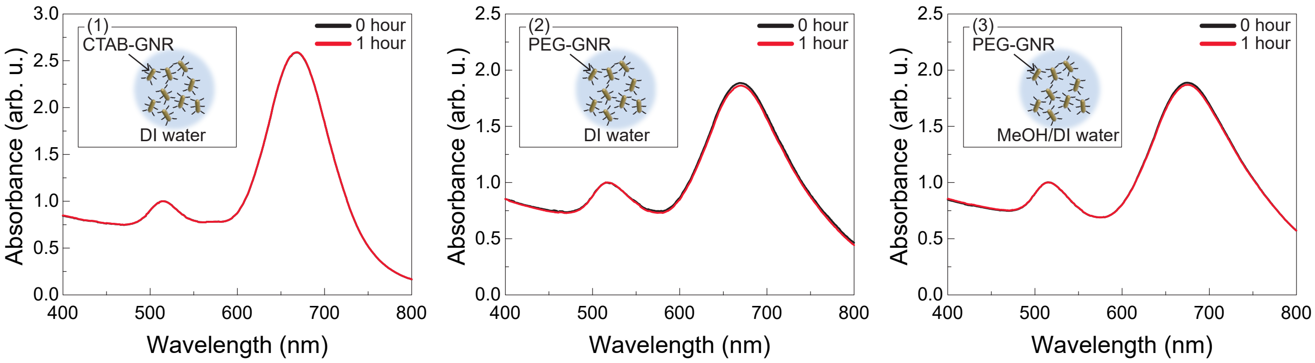

Supplementary Figure 17. Experimental results of UV-Vis absorption spectrum measurement for gold nanorods (GNRs). We performed UV-Vis absorption spectrum measurements on GNRs dispersed under the following three conditions: (1) CTAB-GNR in DI water, (2) PEG-GNR in DI water, and (3) PEG-GNR in a mixture of 70% MeOH and 30% DI water by volume ratio. The black line represents the initial measurement, and the red line depicts the results taken after 1 hour. All measured data were normalized with respect to the peak at a wavelength of 515 nm. Source data are provided as a Source Data file.

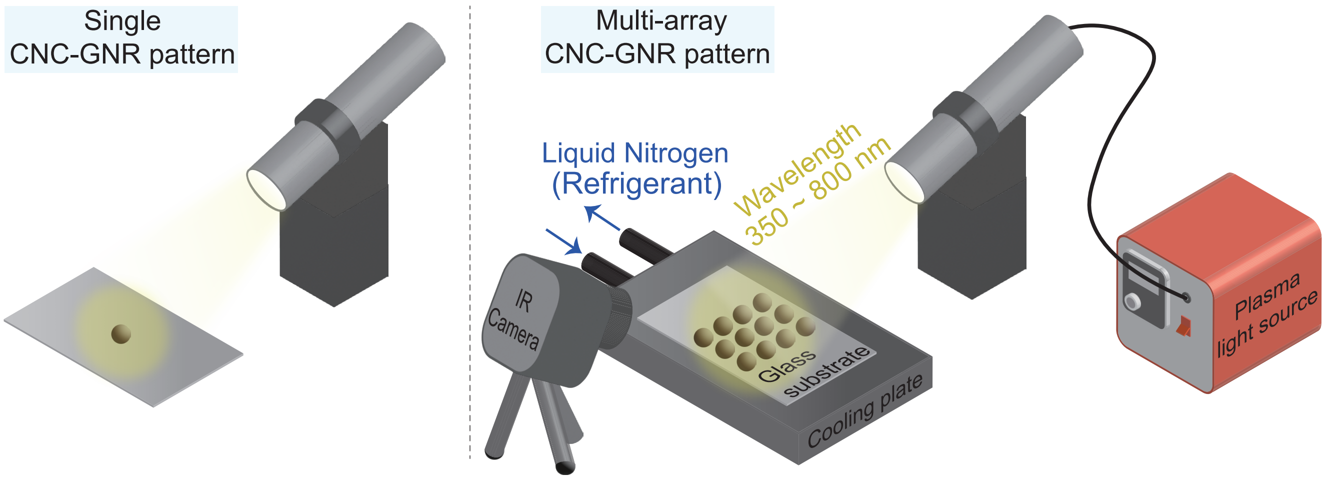

Supplementary Figure 18. Schematic of the experimental setup for generating plasmonic photothermal effects. We deposited CNC-GNR dot films on a glass substrate. To observe the plasmonic photothermal effect of the single CNC-GNR pattern (left) and the multi-array CNC-GNR pattern (right), we irradiated them with a plasma light (wavelength range: 350 - 800 nm) and captured a snapshot with an infrared camera (see the detailed procedure in the Method section). The single and multi-array CNC-GNR patterns were evaluated under different substrate temperatures $T_{sub.}$. The substrate temperature for the single pattern was approximately 26 °C (left), while for the multi-array pattern, it ranged from approximately 22 °C to -8 °C (right). In the multi-array system, the substrate temperature $T_{sub.}$ was regulated by using a cooling plate placed under the CNC-GNR films (see operational principles in the Method section).
