# Supplementary material for: Plasmonic metasurfaces of cellulose nanocrystal matrices with quadrants of aligned gold nanorods for photothermal anti-icing
Source: Nat Commun. 2023 Dec 8;14:8096. doi: 10.1038/s41467-023-43511-9 (PMC10709361; doi:10.1038/s41467-023-43511-9)
Supplement: Supplementary file 3 — Description of Additional Supplementary Files [file 41467_2023_43511_MOESM3_ESM.pdf]

Description of Additional Supplementary Files

File Name: Supplementary Movie 1
Description: A real-time POM Movie for evaporation of the MeOH 0% and DI water 100% droplet with 2.85 wt% CNCs

File Name: Supplementary Movie 2
Description: A real-time POM Movie for evaporation of the MeOH 30% and DI water 70% droplet with 2.85 wt% CNCs

File Name: Supplementary Movie 3
Description: A real-time POM Movie for evaporation of the MeOH 70% and DI water 30% droplet with 2.85 wt% CNCs

File Name: Supplementary Movie 4
Description: A real-time POM Movie for evaporation of the MeOH 70% and DI water 30% droplet with 3.40 wt% CNCs

File Name: Supplementary Movie 5
Description: Side view Movie for evaporation of the MeOH 70% and DI water 30% droplet with 3.40 wt% CNCs

File Name: Supplementary Movie 6
Description: Micro-PIV Movie for evaporation of the MeOH 70% and DI water 30% droplet with 3.40 wt% CNCs

File Name: Supplementary Movie 7
Description: Micro-PIV Movie for evaporation of the MeOH 70% and DI water 30% droplet with 3.40 wt% CNCs and 0.28 wt% GNRs

File Name: Supplementary Movie 8
Description: CNC-GNR droplet evaporation (MeOH : DI water : CNCs : GNRs = 62.57 : 33.76 : 3.39 : 0.28 wt%)

File Name: Supplementary Movie 9
Description: An infrared thermal image for the plasmonic photothermal effect of ring-shaped CNC-GNR films ($C_{GNR} \approx 0.28$ wt%)

File Name: Supplementary Movie 10
Description: An infrared thermal image for the plasmonic photothermal effect of uniform CNC-GNR films ($C_{GNR} \approx 0.28$ wt%)

File Name: Supplementary Movie 11
Description: An infrared thermal image for the plasmonic photothermal effect of uniform CNC-GNR films ($C_{GNR} \approx 0.56$ wt%)

File Name: Supplementary Movie 12
Description: Anti-icing experiment Movie with a 4 × 3 multi-array CNC-GNR film under substrate temperature $-8^{\circ}C$

File Name: Supplementary Movie 13
Description: De-icing experiment Movie with a 4 × 3 multi-array CNC-GNR film under substrate temperature $-8^{\circ}C$
